# The effects of a hydrolyzed protein diet on the plasma, fecal and urine metabolome in cats with chronic enteropathy

**DOI:** 10.1038/s41598-023-47334-y

**Published:** 2023-11-15

**Authors:** Aarti Kathrani, Sandi Yen, Edward J. Hall, Jonathan R. Swann

**Affiliations:** 1 Royal Veterinary College, Hawkshead Lane, Hertfordshire, AL9 7TA UK; 2 Oxford Centre for Microbiome Studies, Kennedy Institute of Rheumatology, University of Oxford, Oxford, OX3 7FY UK; 3 Bristol Veterinary School, University of Bristol, Langford, Bristol, BS40 5DU UK; 4 School of Human Development and Health, Faculty of Medicine, University of Southampton, Southampton, SO16 6YD UK; 5 Department of Metabolism, Digestion, and Reproduction, Imperial College London, London, SW7 2AZ UK

**Keywords:** Metabolomics, Gastroenterology

## Abstract

Hydrolyzed protein diets are extensively used to treat chronic enteropathy (CE) in cats. However, the biochemical effects of such a diet on feline CE have not been characterized. In this study an untargeted ^1^H nuclear magnetic resonance spectroscopy-based metabolomic approach was used to compare the urinary, plasma, and fecal metabolic phenotypes of cats with CE to control cats with no gastrointestinal signs recruited at the Royal Veterinary College (RVC). In addition, the biomolecular consequences of a hydrolyzed protein diet in cats with CE was also separately determined in cats recruited from the RVC (n = 16) and the University of Bristol (n = 24) and whether these responses differed between dietary responders and non-responders. Here, plasma metabolites related to energy and amino acid metabolism significantly varied between CE and control cats in the RVC cohort. The hydrolyzed protein diet modulated the urinary metabolome of cats with CE (*p* = 0.005) in both the RVC and Bristol cohort. In the RVC cohort, the urinary excretion of phenylacetylglutamine, *p*-cresyl-sulfate, creatinine and taurine at diagnosis was predictive of dietary response (*p* = 0.025) although this was not observed in the Bristol cohort. Conversely, in the Bristol cohort plasma betaine, glycerol, glutamine and alanine at diagnosis was predictive of outcome (*p* = 0.001), but these same results were not observed in the RVC cohort. The biochemical signature of feline CE in the RVC cohort was consistent with that identified in human and animal models of inflammatory bowel disease. The hydrolyzed protein diet had the same effect on the urinary metabolome of cats with CE at both sites. However, biomarkers that were predictive of dietary response at diagnosis differed between the 2 sites. This may be due to differences in disease severity, disease heterogeneity, factors unrelated to the disease or small sample size at both sites. As such, further studies utilizing larger number of cats are needed to corroborate these findings.

## Introduction

Feline chronic enteropathy (CE) describes a group of disorders resulting in chronic gastrointestinal signs in the absence of a known cause, such as extraintestinal, infectious, obstructive or localized neoplastic intestinal disease^1, 2^. Unfortunately, there is currently no uniform classification system for feline chronic enteropathy^2^. However, most veterinary gastroenterologists subclassify CE into food-responsive enteropathy (FRE), steroid-responsive enteropathy (SRE) and small cell lymphoma^1, 3–7^. Unfortunately, the pathogenesis of FRE and SRE is unclear and both conditions are diagnosed retrospectively based on treatment response ^1, 2^. However, some cats fail to respond to treatment and are classified as having a non-responsive enteropathy^1, 8^. Despite FRE being common in cats with CE^9^, the ability to differentiate these cats from cats with other enteropathies, such as SRE and non-responsive enteropathy at diagnosis is not possible at this time. As such, there is a need to further elucidate the pathogenesis of CE in cats, to allow for the differentiation of the different subtypes at diagnosis and to enable the development of treatments that are more effective.

Metabolomics is a powerful systems biology approach that simultaneously measures a broad range of low-molecular weight compounds in a biological sample, capturing the metabolic profile or phenotype. This includes metabolites arising from endogenous metabolism and those derived from environmental sources, such as the diet and the gut microbiota^10^. There has been a paucity of metabolomic studies performed in dogs and cats with CE. One study observed that dogs with CE had lower circulating amino acids compared to their healthy equivalents, which may have implications in the dietary management of this disease^11^. In addition, a preliminary study indicated that feces from dogs with CE contained many significantly altered metabolites, including those involved in bile acid and tryptophan metabolism and the pentose phosphate pathway^12^. Similarly, an untargeted metabolomic analysis demonstrated 84 compounds in the feces of cats with CE that differed from those of healthy cats, including metabolites related to tryptophan, arachidonic acid and glutathione pathways^13^. Therefore, metabolomics can provide important insights into disease pathogenesis as well as identify potential targets for therapy.

Metabolic phenotyping is also able to differentiate Crohn’s disease from ulcerative colitis, two types of inflammatory bowel disease (IBD) in humans, and from healthy controls^14^. Interestingly, patients with coeliac disease have a distinct metabolic profile that allows differentiation from healthy patients and this profile reverts to a “healthy” phenotype after 12 months of a strict gluten-free diet^14^. This highlights the ability of metabolomics to differentiate between different subcategories of disease, monitor therapeutic outcomes, and identify diagnostic biomarkers. Therefore, metabolomics may help to characterize the biochemical alterations associated with FRE in cats and whether such perturbations are modulated after a positive dietary response. This information can also help to understand further the biomolecular mechanisms involved in this disorder providing detailed information to guide the development of future dietary interventions. Ultimately such testing should allow differentiation of cats with FRE from cats with other enteropathies. Hydrolyzed protein diets, as well as other categories of diets, such as limited ingredient novel protein, highly digestible and fiber enriched diets have all been used successfully for the treatment of CE in cats^15–22^. However, the metabolomic effects of a hydrolyzed protein diet in cats with CE was the focus of this study, as this category of diets is frequently used for CE in cats^23^.

This study aimed to (1) compare the plasma, fecal and urine metabolome of cats with CE to control cats with no gastrointestinal signs and (2) determine the effect of a hydrolyzed protein diet on the plasma, fecal and urine metabolome of cats with CE and whether this differs between dietary responders and non-responders, using an untargeted ^1^H nuclear magnetic resonance (NMR) spectroscopy-based approach.

## Materials and methods

### Recruitment of case and control cats

Recruitment of case and control cats was the same as described for a previous study performed by the same authors^24^. Cats referred to the Royal Veterinary College (RVC) and University of Bristol for persistent or intermittent GI signs (vomiting and/or diarrhea) of at least 2 weeks duration and where CE was suspected based on thorough investigation were considered for the study. A two week duration of signs was chosen as this was in agreement with previous studies assessing the effects of diet in cats with idiopathic gastrointestinal signs^15, 16, 18, 23–25^. All cats underwent the same diagnostic investigations consisting of a minimum of complete blood count, serum biochemistry with electrolytes, serum cobalamin (with or without folate) concentration and trans-abdominal ultrasound examination. Feline pancreatic lipase immunoreactivity (PLI, *n* = 16), trypsin-like immunoreactivity (TLI, *n* = 25), serum thyroxine (total T4, *n* = 19), pre- or pre and post-prandial bile acid (*n* = 16) concentrations, fecal parasitology using saturated zinc sulfate flotation (*n* = 18), fecal culture (for *Salmonella, Campylobacter* and *Clostridium difficile, n* = 9), empirical deworming (*n* = 10), polymerase chain reaction for *Tritrichomonas foetus* (*n* = 15) and feline leukemia and feline immunodeficiency virus testing (*n* = 14) were performed in some cats as indicated by the history, physical examination and ultrasound examination findings. Cats that had histopathologic confirmation of CE via intestinal biopsy specimens collected via endoscopy or exploratory laparotomy were included as confirmed CE (n = 13), whereas those that had no intestinal biopsies performed, but investigations described above revealed no underlying cause for the chronic GI signs were included as suspected CE (n = 27). Cats where dietary intervention without antimicrobials and immunosuppressant medication was used for treatment were then enrolled. Cats diagnosed with small cell lymphoma on intestinal biopsy specimens were excluded from the study, as this would have necessitated use of additional medications (prednisolone + /- chlorambucil) and therefore response to dietary intervention in the absence of immunosuppressants would not have been able to be assessed. The feline chronic enteropathy activity index (FCEAI) was calculated for all cats with suspected or confirmed CE^4^. For those cats that did not have endoscopy performed, a score of 0 was given for the variable endoscopic lesions^4^. Adult cats (at least 1 year of age) without a history of GI signs and owned by staff members (n = 10) or part of the RVC blood donor program (n = 4) were recruited as controls. All owners were questioned regarding the absence of current and historical Gl signs (vomiting and diarrhea). Unfortunately, longitudinal weight history, full physical examination and laboratory blood work was not performed in these cats to ensure subclinical GI disease was not present nor was a full history collected to rule out other diseases or current medications.

The Royal Veterinary College and University of Bristol granted ethical approval for the study (URN 2018 1837-3 and VIN/14/017, respectively). Written informed consent for participation into the study was obtained from all owners of cats. All methods were carried out in accordance with the research guidelines at the Royal Veterinary College and University of Bristol and are reported in accordance with ARRIVE guidelines.

### Sample collection

For each cat with suspected or confirmed CE, 1 ml of residual blood from diagnostic investigations was directly placed into heparin tubes, the plasma separated and stored at − 80 °C. Naturally voided feces were collected during hospitalization and frozen immediately. Residual urine, either naturally voided onto non-absorbent beads placed in the litter tray or collected via cystocentesis (blind or ultrasound guided) from planned urinalysis was collected and frozen immediately.

For the control cats, 1 ml of residual blood was collected from planned laboratory tests from blood donor cats for screening (n = 4) or staff owned cats for annual wellness check (n = 4) and placed directly into heparin, with plasma extracted and frozen immediately. For all staff owned cats, naturally voided feces and urine was collected at home, using the same brand of non-absorbent beads in the litter tray (n = 9 and n = 8, respectively). Feces and urine were frozen immediately and brought to the referral practice using ice packs.

### Dietary recommendations for cats with CE

Dietary recommendation for cats with CE was the same as described for a previous study performed by the same authors^24^. All cats with suspected or confirmed CE were discharged from the RVC and University of Bristol with the same therapeutic hydrolyzed protein diet.* All owners were instructed to feed the therapeutic diet exclusively for 6 weeks and were specifically instructed against feeding treats and other foods. However, owners were not specifically instructed against the use of oral flavored medication, such as deworming medication or toothpaste. Owners were asked to follow the feeding instructions on the pet food label according to current body weight and to adjust daily calories to maintain current body weight and condition if the cat was at an ideal condition or over-conditioned and to achieve and then maintain an ideal body condition if the body condition score was 3/9 or below^26^. Control cats were not subjected to any dietary intervention.

### Sample collection post dietary intervention

The owners of all cats with suspected or confirmed CE were asked to schedule a recheck appointment after receiving the hydrolyzed protein diet for 6 weeks. For those cats that returned (n = 12), owners brought naturally voided feces and urine to the appointment, which were frozen immediately in separate vials and transported on ice packs to the referral hospital. Blood was collected via jugular venipuncture for repeat serum biochemistry and 1 ml of residual placed directly into heparin, the plasma separated and stored at − 80 °C. For those cats that were unable to return (n = 28), but owners were able to collect feces and urine at home, samples were immediately stored in multiple plastic bags in the freezer at the owners’ homes and transported to their local veterinary practice on ice. All samples were transported back to the referral hospital on dry ice. All fecal and urine samples were stored at − 80 °C until analysis.

### Assessing response to hydrolyzed protein diet in cats with CE

Assessment of response to the hydrolyzed protein diet in cats with CE was the same as described for a previous study performed by the same authors^24^. Owners of cats with suspected or confirmed CE were contacted via email, telephone or in person at their scheduled 6 week recheck visit to assess the response of the cat to the hydrolyzed protein diet after 6 weeks of feeding. Responders were those cats where the owners reported a significant reduction in GI signs, which did not warrant any change or additional therapy. Non-responders included those cats that were reported to have no or partial response necessitating discontinuation of the hydrolyzed protein diet with a change to a different therapeutic diet or continuation of the hydrolyzed protein diet with the addition of prednisolone, or further diagnostic investigations, such as GI biopsy if not previously performed.

### ^1^H nuclear magnetic resonance (NMR) spectroscopy-based metabolomic analysis

Metabolic profiles were measured by untargeted ^1^H NMR spectroscopy using standard protocols. Samples from the RVC and Bristol cohort were processed and analyzed separately. All spectra were acquired on a 600 MHz Bruker Avance III spectrometer equipped with a refrigerated SampleJet autosampler maintained at 6 °C (Bruker Biospin GmbH, Rheinstetten, Germany). Urine samples were prepared by combining 630 μl of sample with 70 μl of phosphate buffer solution (pH 7.4, 100% D_2_O) containing 1 mM of the internal standard, 3-trimethylsilyl-1-[2,2,3,3-2H4] propionate (TSP). Samples were vortexed to mix, spun at 10,000 *g* and the supernatant was transferred to 5 mm NMR tubes. Fecal water was prepared by combining 50 mg of fecal material with 700 μl of demineralized water and ~ 10 zirconium beads. Samples were homogenized using a Precellys 24 homogenizer (Bertin Instruments, France) with 2 × 6,500 rpm cycles (2 × 40 s homogenization, followed by 20 s interval). Following homogenization, samples were spun at 10,000 *g* for 20 min at 4 °C and the supernatant (630 μl) was removed, combined with 70 μl of phosphate buffer, and transferred to a 5 mm NMR tube. Plasma samples (300 μl) were combined with 300 μl of plasma buffer (0.075 M NaH_2_PO_4_, 2 mM NaN_3_, 100% D_2_O, pH 7.4), vortexed to mix and spun at 10,000 *g* for 10 min before transfer to 5 mm NMR tubes. Fecal and urine spectra were acquired using a standard one-dimensional solvent suppression pulse sequence (relaxation delay, 90° pulse, 4-μs delay, 90° pulse, mixing time, 90° pulse, acquire free induction decay). Each spectrum was acquired with 32 scans, 4 dummy scans, 64,000 frequency domain points and a spectral window set to 20 ppm (parts per million). A Carr-Purcell-Meiboom-Gill (CPMG) experiment with water suppression was used to measure the plasma samples (32 scans, 4 dummy scans, 64,000 domain points, 20 ppm spectral window). All spectra were automatically phase and baseline corrected in Topspin 3.2 (Bruker Biospin GmbH, Rheinstetten, Germany). Urine and fecal spectra were referenced to the TSP resonance at δ 0.0, and the plasma spectra were referenced to the glucose resonance at δ 5.22. The raw spectra were imported into Matlab (version 2018a, MathWorks Inc.) and aligned where necessary using the Imperial Metabolic Profiling and Chemometrics Toolbox (https://github.com/csmsoftware/IMPaCTS). The urine and fecal spectra were normalized to minimize sample dilution effects using a total area approach.

### Data analysis

Orthogonal projection to latent structures-discriminant analysis (OPLS-DA) models were built to identify metabolic features associated with variables of interest (e.g., CE versus control, pre-diet versus post-diet, responders to diet versus non-responders at diagnosis and post-diet). Here, the ^1^H NMR metabolic profiles served as the descriptor matrix and disease status, time, or dietary response served as the response variable. The predictive power (Q^2^Y) of each model was assessed using a seven-fold cross-validation approach and model validity (provided as *P* value) was calculated through permutation testing (1,000 permutations; significance for *P*_*per*_ ≤ 0.05). When sample numbers prevented the use of OPLS-DA, unsupervised principal components analysis (PCA) models were built ^27, 28^.

When comparing the metabolic profiles of cats with CE versus controls, only cats with CE recruited from the RVC were used in the analysis. When assessing the effects of the hydrolyzed protein diet on the metabolome and comparing responders versus non-responders pre and post diet, cats recruited from the RVC and University of Bristol were analyzed separately. Not all cats had plasma, urine and feces collected at both time points. Therefore, the number of cats that had comparisons performed varied and is depicted in Table 1.

**Table 1:** Number of cats recruited from the two referral veterinary hospitals and number of cats included in the different comparisons of metabolites. CE: chronic enteropathy, RVC: Royal Veterinary College.

		RVC cohort	Bristol cohort
Total number of cats recruited	CE	16	24
	Controls	10	4
CE vs. Controls	Plasma	16 vs. 8	–
	Urine	12 vs. 4	–
	Feces	14 vs. 8	–
Pre vs. Post diet (CE only)	Plasma	–	22 vs. 11
	Urine	12 vs. 11	20 vs. 12
	Feces	13 vs. 11	22 vs. 12
Pre diet; responder vs. non-responder (CE only)	Plasma	8 vs. 6	9 vs. 14
	Urine	6 vs. 4	7 vs. 13
	Feces	6 vs. 7	8 vs. 14
Post diet; responder vs. non-responder (CE only)	Plasma	–	5 vs. 6
	Urine	6 vs. 5	7 vs. 5
	Feces	7 vs. 4	7 vs. 5

The Mann–Whitney U test was used to determine if there was a significant difference in FCEAI and age between cats with CE recruited from the RVC and those recruited from the University of Bristol. The Mann–Whitney U test and Fisher’s exact test was used to determine if there was a significant difference between age and sex/neuter status and breed, respectively between cats with CE recruited at the RVC and controls. Finally, the Mann–Whitney U test was used to determine if there were any significant differences in FCEAI and duration of gastrointestinal signs between those cats that responded to the hydrolyzed protein diet and those that did not. All statistical analysis was performed using Statistical Package for Social Sciences Version 26.

### Ethics approval and consent to participate

The Royal Veterinary College and University of Bristol granted ethical approval for the study (URN 2018 1837-3 and VIN/14/017, respectively).

## Results

### RVC cohort

Cats with confirmed or suspected CE (*n* = 16) were prospectively enrolled in the study at the RVC. The CE group included 10 neutered males and 6 neutered females. The age of the cats ranged from 2 to 17 years, with a median age of 6.5 years. Ten cats were domestic shorthair, 2 domestic longhair, 2 Bengal, 1 Ragdoll and 1 Burmese. Body condition score ranged from 2/9 to 7/9, with a median of 5/9. The duration of GI signs for all cats in the CE group ranged between 1 to 120 months (median, 6 months).

Intestinal biopsies were performed in five cats. Lymphoplasmacytic enteritis was observed in 2 cats, and lymphoplasmacytic and neutrophilic enteritis was noted in the other 3 cats. No cases of small cell lymphoma were diagnosed. The median FCEAI for all cats was 5.5, with a range of 2–10.

### Bristol cohort

Cats with confirmed or suspected CE (*n* = 24) were prospectively enrolled in the study at the University of Bristol. The CE group included 17 neutered males, 6 neutered females and 1 intact female. The age of the cats ranged from 9 months to 19 years, with a median age of 7.5 years. Ten cats were domestic shorthair, 3 domestic longhair, 3 Siamese, 2 Bengal, 2 Ragdoll, 2 Maine Coon, 1 Abyssinian and 1 British Shorthair. Body condition score ranged from 3/9 to 7/9, with a median of 4/9. The duration of GI signs for all cats in the CE group ranged between 0.5 month to 78 months (median, 3 months).

Intestinal biopsies were performed in 9 cats. Lymphoplasmacytic and eosinophilic enteritis was observed in 4 cats, and one each had the following histopathology: mixed enteritis, lymphoplasmacytic enteritis, lymphoplasmacytic and neutrophilic colitis and no abnormalities detected. One cat was diagnosed with small cell lymphoma and was excluded. The median FCEAI for all eligible cats was 3, with a range of 1–9.

The clinical severity of disease, as assessed by FCEAI was significantly higher for the RVC cohort compared to the Bristol cohort (p = 0.011). There was no significant difference in age between the RVC and Bristol cohorts (p = 0.859). There were no significant differences in FCEAI and duration of gastrointestinal signs between cats that responded to the hydrolyzed protein diet and those that did not (p = 0.421 and 0.950, respectively).

### Control cats

The control cats consisted of 10 staff owned cats and 4 blood donor cats. This included 9 male neutered, 4 female neutered and 1 female intact. The age of the cats ranged from 1 to 15 years with a median of 7 years. Breeds included 5 Domestic shorthair, 5 Domestic longhair and one each of the following breeds: British shorthair, Maine Coon, Russian Blue and Bengal. No control cats had a previous or current history of GI signs.

There were no significant differences in age, sex/neuter status, and breed between cats with CE (RVC cohort) and control cats (*p* > 0.468).

### Metabolomic profiling by ^1^H NMR spectroscopy of CE cats (RVC cohort) and control cats

**Figure 1:**
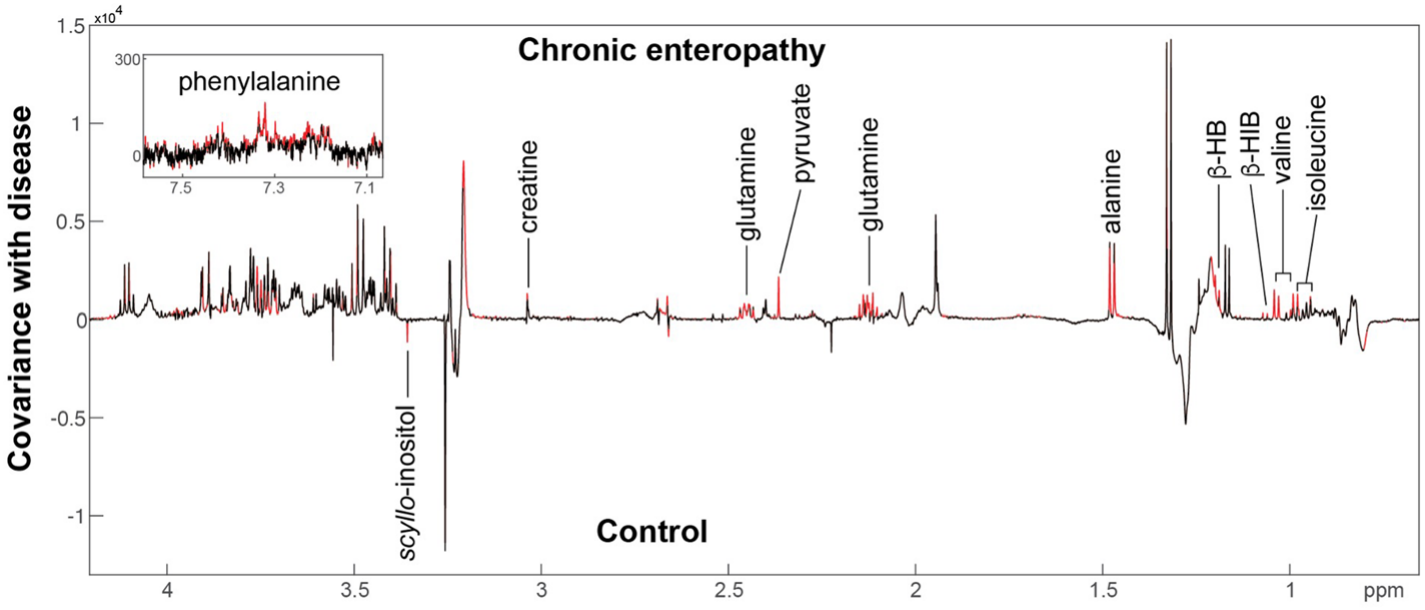
OPLSDA model comparing the plasma metabolic profiles of control cats and those with chronic enteropathy enrolled at the RVC. Red peaks indicate those with a significant correlation (*p* < 0.05) with disease status. β-HB, β-hydroxybutyrate; β-HIB, β-hydroxyisobutyrate.

The metabolomes of plasma, fecal and urine samples collected at the time of diagnosis of CE from cats recruited at the RVC were compared to control cats. OPLS-DA models were built to compare the CE (*n* = 16) and control plasma profiles (*n* = 8). A model with significant predictive ability (Fig. 1; Q^2^Y = 0.22; *p* = 0.04) was obtained indicating biochemical variation in the plasma between the two animal groups. The CE plasma contained higher amounts of the amino acids, alanine, glutamine, valine, isoleucine, and phenylalanine, as well as higher amounts of pyruvate, β-hydroxybutyrate, β-hydroxyisobutyrate and creatine compared to the control cats. Conversely, control plasma had higher circulating amounts of *scyllo-*inositol.

The OPLS-DA model comparing the fecal metabolic profiles of control (*n* = 8) and CE cats (*n* = 14) had poor predictive ability (Q^2^Y = − 0.295). Due to the small size of the control group (*n* = 4) in the urine dataset, an unsupervised PCA model was constructed to compare these control profiles to the CE group (n = 12). No separation was observed between the groups (Supplementary Fig. 1).

### Effect of the hydrolyzed protein diet on the metabolome of cats with CE—RVC cohort

**Figure 2:**
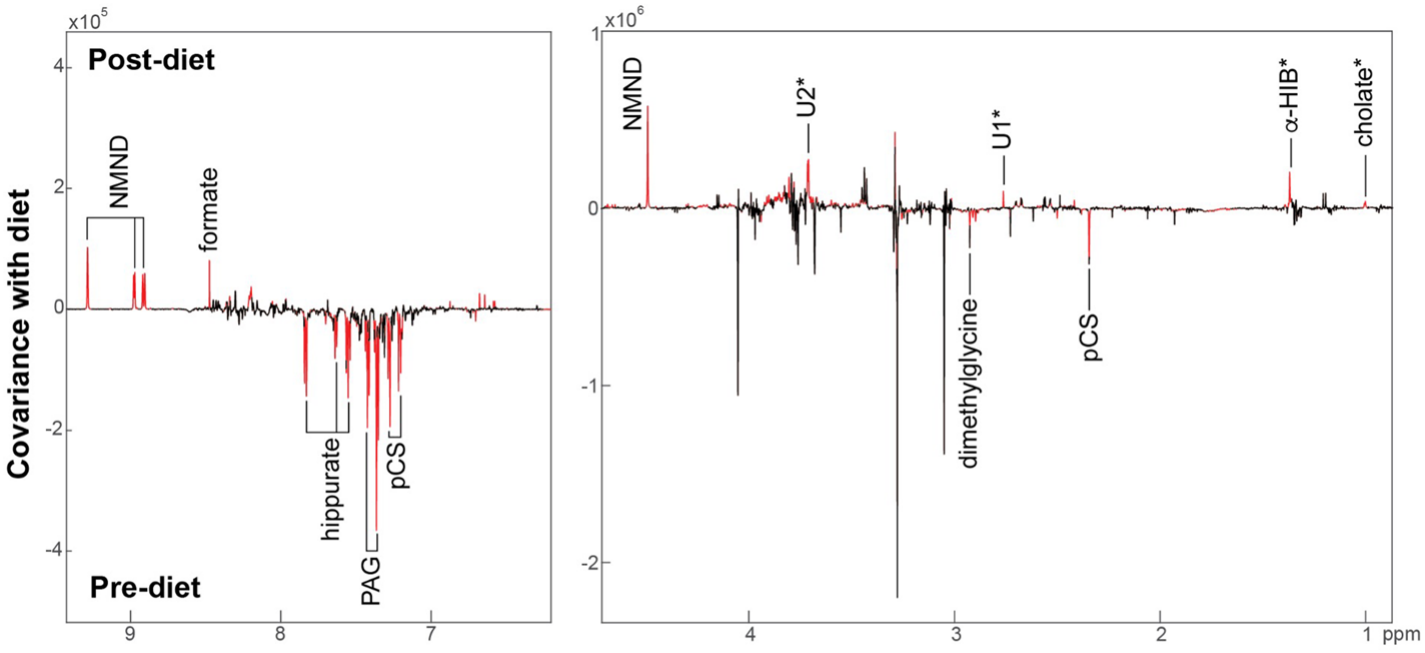
OPLSDA model comparing the urinary metabolic profiles of cats with chronic enteropathy before and after receiving a hydrolyzed protein diet for the RVC cohort. Red peaks indicate those with a significant correlation (*p* < 0.05) with sampling point. α-HIB, α-hydroxyisobutyrate; NMND, *N*-methylnicotinamide; PAG, phenylacetylglutamine; pCS, *p*-cresyl-sulfate; U1-U2, Unknown metabolite 1–2.

A significant OPLS-DA model was obtained comparing the urinary metabolic profiles of cats with CE before and after receiving the hydrolyzed protein diet (Fig. 2**;** Q^2^Y = 0.336; *p* = 0.005). From this model the diet was shown to increase the urinary excretion of *N*-methylnicotinamide (NMND), formate, α-hydroxyisobutyrate and cholate and decrease the excretion of the microbial-host co-metabolites hippurate, phenylacetylglutamine (PAG), and *p-*cresyl-sulfate (*p*CS) as well as dimethylglycine (DMG). No diet-related differences were observed in the fecal profiles and no post-diet plasma samples were collected for this cohort.

### Effect of the hydrolyzed protein diet on the metabolome of cats with CE—Bristol cohort

A significant model (Q^2^Y = 0.337; *p* = 0.002) was obtained comparing the urinary profiles of CE cats pre-diet (*n* = 20) and post-diet (*n* = 12) from cats in the Bristol cohort. The OPLS-DA coefficients plot indicated that the diet increased urinary NMND, taurine, methionine, acetate, and *N*-acetylglycoproteins, and decreased hippurate and PAG (Supplementary Fig. 2A). A similar model was obtained considering only matched samples at each time point (12 cats; Q^2^Y = 0.239; *p* = 0.021). The OPLS-DA model comparing the fecal signatures pre- (*n* = 22) and post-diet (*n* = 12) was also significant (Q^2^Y = − 0.206; p = 0.001) with the hydrolyzed protein diet observed to increase the gut microbial metabolites, butyrate, lactate, and acetate (Supplementary Fig. 2B). The OPLS-DA model comparing plasma pre- and post-diet was not significant (Q^2^Y = − 0.036).

### Pre-diet, responders versus non-responders at diagnosis-RVC cohort

**Figure 3:**
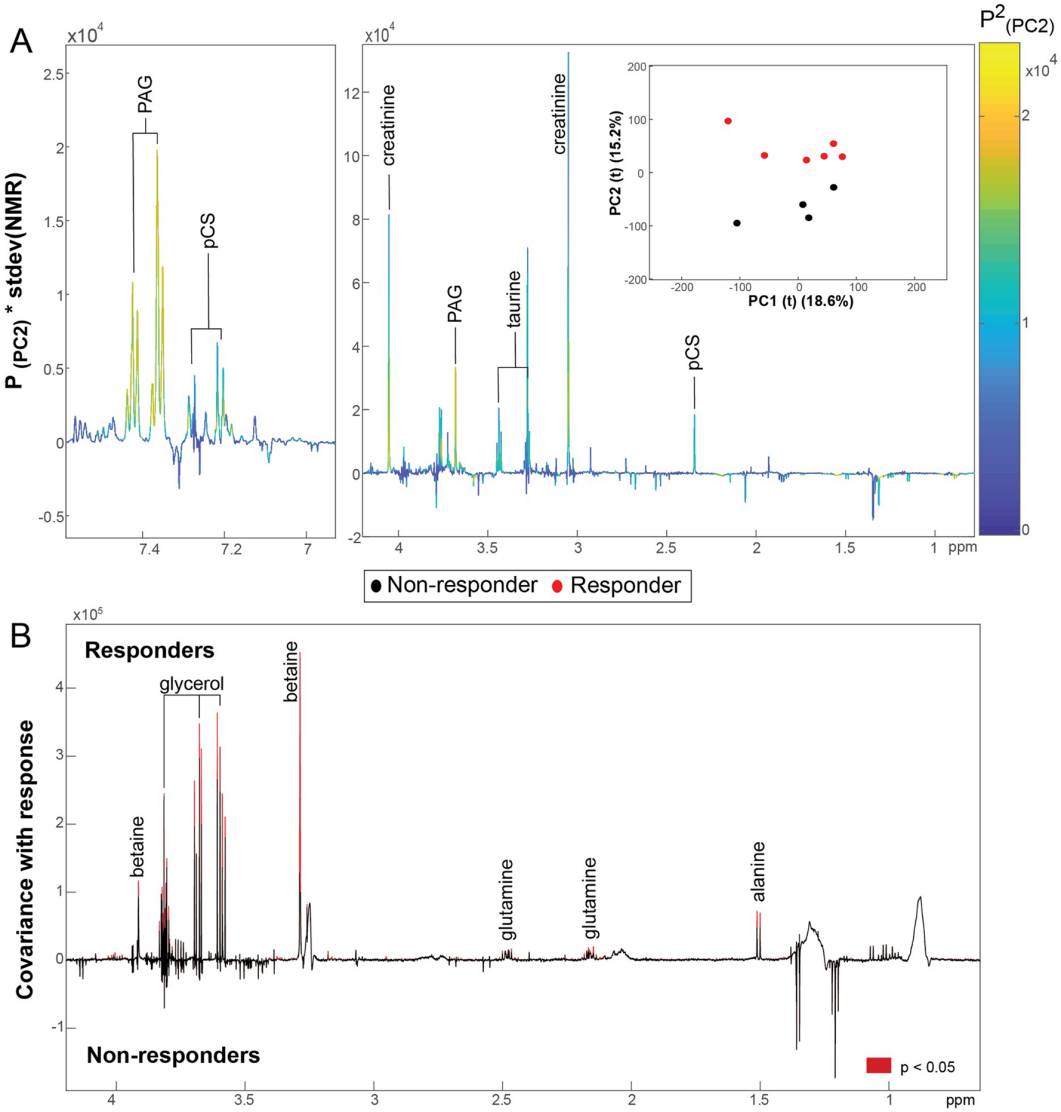
Biochemical predictors of response to hydrolyzed protein diet. (**A**) PCA model comparing the pre-diet urinary metabolic phenotypes of cats with CE that responded and did not respond to the hydrolyzed protein diet (RVC cohort). Scores plot shows separation along the second principal component (PC2) based on response status. The loadings plot for PC2 highlights that responders excrete greater amounts of creatinine, PAG, pCS, taurine. (**B**) OPLS-DA model comparing the pre-diet plasma metabolic profiles of CE cats from the Bristol cohort that responded and did not respond to the hydrolyzed protein diet (Q^2^Y = 0.507; *p* = 0.001). Red peaks indicate those with a significant correlation (*p* < 0.05) with diet response. PAG, phenylacetylglutamine; pCS, *p*-cresyl-sulfate.

Next, the metabolic signatures of cats with CE were explored to identify biochemical features at baseline that could predict whether a cat would respond to the diet. An unsupervised PCA model was constructed on the pre-diet urinary metabolomes due to the small group sizes (6 responders, 4 non-responders). Here, clear separation was observed in the second principal component of the scores plot based on response status (Fig. 3A). From the loadings plot the responders were noted to excrete greater amounts of pCS, PAG, taurine and creatinine compared to the non-responders. pCS and PAG are microbial-host co-metabolites derived from the metabolism of dietary tyrosine and phenylalanine, respectively^29^.

No significant differences were observed between the responders and non-responders in the pre-diet plasma (8 responders, 6 non-responders) or fecal (6 responders, 7 non-responders) profiles of the RVC cohort.

### Pre-diet, responders versus non-responders at diagnosis-Bristol cohort

In the Bristol cohort a significant model was obtained comparing the biochemical signatures of plasma collected prior to receiving the diet between those that responded (*n* = 9) and those that did not (*n* = 14) (Q^2^Y = 0.507; *p* = 0.001; Fig. 3B). Those that responded to the hydrolyzed protein diet had higher circulating amounts of alanine, glutamine, betaine, and glycerol at diagnosis. It was not possible to discriminate between the responders (*n* = 7 urinary, *n* = 8 fecal) and non-responders (*n* = 13 urinary, *n* = 14 fecal) in pre-diet urinary (Q^2^Y = −0.249) and fecal (Q^2^Y = − 0.135) profiles.

### Post-diet, responders versus non-responders

No response-dependent differences were seen in the urinary (6 responders versus 5 non-responders) or fecal (7 responders versus 4 non-responders) metabolomic profiles of cats with CE following the hydrolyzed protein diet in the RVC cohort. No post-diet plasma samples were collected for this cohort. Similarly, no response-dependent differences were seen in the plasma (5 responders versus 6 non-responders), urinary (7 responders versus 5 non-responders) or fecal (7 responders versus 5 non-responders) metabolomic profiles of cats with CE following the hydrolyzed protein diet in the Bristol cohort.

## Discussion

In this study we identified metabolic variation with regards to energy and amino acid metabolites in cats associated with CE. This finding further expands our understanding of the biomolecular mechanisms contributing to the pathogenesis of CE in cats. Such observations may assist with the development of novel diagnostic markers and identify targets for therapeutic interventions. In addition, we observed that a therapeutic hydrolyzed protein diet was able to modulate the urinary metabolome of cats with CE from both the RVC and Bristol cohorts and the fecal metabolomes of cats with CE from the Bristol cohort. Furthermore, our results suggest that the urinary metabolome from the RVC cohort and the plasma metabolome from the Bristol cohort may be predictive of response to a hydrolyzed protein diet in cats with CE at diagnosis. However, our results should be interpreted with caution, due to the low number of cats in some of the responder and non-responder groups. Therefore, these results should be considered preliminary and future studies should aim to utilize larger number of cats to corroborate these findings.

In the RVC cohort, cats with CE were found to have higher circulating amounts of beta-hydroxyisobutyrate, which is the catabolic intermediate of the branched chain amino acid valine^30^ compared to control cats. Disturbed amino acid metabolism has been demonstrated in the serum and plasma of humans with IBD^31^ and dogs and cats with CE^32–34^. In the RVC cohort, plasma concentrations of three amino acids; phenylalanine, isoleucine and alanine were increased in the plasma of cats with CE compared to controls. One study showed increased amounts of phenylalanine in the serum of active IBD patients compared to controls and IBD remission patients and in their study, this was one of the metabolites found to be the strongest candidate as a biomarker for disease^32, 35^. In another study, increased concentrations of serum phenylalanine were seen in dogs with CE compared to controls^32^. The authors of both studies speculated that the increased concentrations were due to the immune response inhibiting phenylalanine-4-hydroxylase activity, the enzyme responsible for hydrolyzing phenylalanine into tyrosine. Elevated plasma phenylalanine may also reflect reduced metabolism of dietary phenylalanine by the intestinal microbiota increasing its bioavailability for the host. Finally, similar to our results from the RVC cohort, previous studies in humans have also documented an increase in serum isoleucine concentration in IBD patients compared to controls^35, 36^.

The conversion of glutamine to alanine provides the intestinal mucosa with approximately one third of the required energy to achieve metabolic demand^37^. Therefore, the increased plasma alanine concentrations could reflect a high demand for energy in the intestinal mucosa of cats with CE^36, 38^. This is also likely exemplified by the increased plasma concentrations of pyruvate seen in these cats, which indicates up-regulation of glycolysis due to higher need for cellular energy or lack of energy sources under inflammatory and immune conditions^39, 40^ and is similar to metabolic alterations seen in human and animal models of IBD^41^.

Our study showed higher plasma *scyllo*-inositol in control cats compared to cats with CE in the RVC cohort. Control mice have higher concentrations of *scyllo*-inositol in their colon compared to mice with colitis^42^. *Scyllo*-inositol is produced by the intestinal microbiota in response to dietary inulin and has mostly been investigated as a therapeutic agent for Alzheimer’s disease^43, 44^. Whether *scyllo*-inositol is protective for the development of feline CE and whether this could be used as a therapeutic agent in this disease warrants further investigation.

Our study showed that a therapeutic hydrolyzed protein diet modulated the urinary metabolome of cats with CE from both cohorts after 6 weeks of exclusive feeding. For both the RVC and Bristol cohorts, the hydrolyzed protein diet increased urinary NMND and decreased urinary PAG and hippurate. NMND is a metabolite of niacin and an indicator of niacin status^45^. Cats have a strict dietary requirement for pre-formed niacin, as they are unable to synthesize this vitamin using the tryptophan-nicotinamide pathway^46^. As such, increased urinary concentrations following intervention with a hydrolyzed protein diet in these cats could be an indicator of improved niacin status. This could occur either due to increased concentrations in the hydrolyzed protein diet, increased digestibility of the diet improving bioavailability of this vitamin or reduced intestinal inflammation resulting in increased absorption of this vitamin.

PAG is a microbial-host co-metabolite produced from the fermentation of phenylalanine to phenylacetate by the intestinal microbiota, which is absorbed and subsequently conjugated with glutamine in the host^47^. The decreased urinary concentrations seen in cats with CE following intervention with the hydrolyzed protein diet in both the RVC and Bristol cohorts most likely reflects improved digestion and absorption of amino acids in the hydrolyzed protein diet in the proximal intestine resulting in less phenylalanine reaching the colon for fermentation. This is also in agreement with the hydrolyzed protein diet decreasing urinary concentrations of *p*-cresyl sulfate in the RVC cohort. *p*-cresyl sulfate is another microbial-host co-metabolite arising from the microbial metabolism of tyrosine and phenylalanine, which is then sulfated by the host^47^. Hydrolyzed protein diets consist of smaller proteins, typically in the range of 10–12 kilodaltons^48^, which improves their digestibility and hence, the amount of amino acids reaching the distal GI tract. Interestingly, the cats that responded to the diet in the RVC cohort excreted greater amounts of PAG and *p*-cresyl sulfate at diagnosis compared to those that did not respond. Further work is necessary to determine the contribution of these pan-kingdom metabolites to intestinal inflammation and the CE phenotype and establish if a distinct phenotype of CE exists that is driven by these microbial metabolites. Growing evidence supports a negative association between intestinal p-cresol and gut health^49, 50^.

Urinary hippurate is a product of the microbial metabolism of certain dietary compounds including phenylalanine^51, 52^. It has previously been shown that hippurate positively correlated with the presence of *Clostridia* *spp.* in the gut^51, 53^. Our study demonstrated that cats with CE in both the RVC and Bristol cohorts had lower amounts of urinary hippurate following intervention with a hydrolyzed protein diet. Interestingly, previous studies have shown increased fecal Clostridia in cats with CE^24, 54^. Therefore, the hydrolyzed protein diet could positively modulate the hippurate-associated bacteria in the gut, like Clostridia. Further studies should ascertain the association, if any, between intestinal C*lostridia spp.* and urinary hippurate concentrations in cats with CE.

Our study demonstrated that urinary biomarkers at diagnosis may be able to predict whether cats with CE will respond to intervention with a hydrolyzed protein diet in the RVC cohort. However, it is important to note that the number of cats utilized in these comparisons were small. Therefore, our results are suggestive and would need studies utilizing larger number of cats to corroborate these findings and to ensure any significant differences seen in our study are not due to a type 1 error from small sample numbers. However, our preliminary results are interesting, as dietary responders had higher urinary concentrations of phenylacetylglutamine, *p*-cresyl sulfate and creatinine compared to non-responders at diagnosis. Given that the hydrolyzed protein diet was shown to reduce urinary concentrations of phenylacetylglutamine in both the RVC and Bristol cohorts and urinary *p*-cresyl sulfate in the RVC cohort, it is not surprising that cats with CE and higher urinary concentrations of these two metabolites at diagnosis are more likely to benefit from a diet that is able to decrease the concentrations of both. That these cats responded clinically when the concentrations of both metabolites decreased highlights the importance and role of phenylacetylglutamine and *p*-cresyl sulfate in the pathogenesis of a subset of cats with CE. Interestingly, both phenylacetylglutamine and *p*-cresyl sulfate are described as gut-derived uremic toxins^47^. Therefore, this could suggest a role for the gut-kidney axis in some cats with food-responsive enteropathy, especially as creatinine, a metabolite commonly associated with kidney function^55^, was also increased in the urine at diagnosis in dietary responders compared to non-responders. Given that the hydrolyzed protein diet reduced both gut-derived uremic toxins, further studies assessing increased digestibility and the role of these diets in feline kidney disease may be justified. Additionally, the direct role of gut-derived uremic toxins on the GI tract and the exact mechanism of action of hydrolyzed protein diets as treatment in these cats necessitates further investigation. The results of such investigations could also help to provide insight into the pathogenesis of human IBD.

Our study showed that plasma biomarkers at diagnosis may be able to predict whether cats with CE will respond to intervention with a hydrolyzed protein diet in the Bristol cohort. As this was different to the results obtained from the RVC cohort, this suggests that cats with food-responsive enteropathy may be heterogenous with regards to disease pathomechanism, with the same diet being able to target these various perturbations leading to clinical response. In addition, the cats from the Bristol cohort had significantly lower disease severity scores compared to the RVC cohort, which may also explain the difference in results between the two cohorts. However, it is also important to note that as our comparison of dietary responders and non-responders contained a small number of cats at both sites, this could have reduced statistical power leading to a lack of significance in plasma biomarkers across centers. Future studies utilizing larger number of cats will help to determine if the difference in results between centers is due to disease heterogeneity, severity scores or due to reduced statistical power from a type II error. Additionally, differences between the two cohorts might also be explained by factors not related to the disease, such as differences in the environment, nature of sample collection, or the way the data was analyzed.

Although, our results are preliminary and suggestive, they showed that dietary responders in the Bristol cohort had higher plasma concentrations of betaine, glycerol, glutamine and alanine compared to dietary non-responders at diagnosis. Interestingly, most of these metabolites (betaine, glycerol and glutamine) have been shown to ameliorate or decrease gastrointestinal inflammation in animal models of IBD^56–58^ and plasma betaine and glutamine have been shown to be decreased in humans with IBD^36, 59^. Therefore, their increased presence in dietary responders at diagnosis could represent adaptive mechanisms already at play for reducing intestinal inflammation, which could also explain their significantly lower disease severity scores. As these cats are likely already undergoing compensatory mechanisms to reduce intestinal inflammation, intervention with a hydrolyzed protein diet alone might be sufficient for inducing remission negating the need for glucocorticoids. Interestingly, the diet increased the fecal excretion of the gut microbial metabolites, butyrate, acetate and lactate only in this cohort of cats. All three of these metabolites have been shown to reduce or prevent intestinal inflammation in experimental models of IBD^60–62^. Therefore, the increase of these additional anti-inflammatory metabolites in cats already undergoing compensatory mechanisms to reduce intestinal inflammation might be enough to induce remission.

Higher concentrations of betaine and glutamine in dietary responders at diagnosis may suggest less severe disease where dietary treatment alone may be adequate at inducing remission. Interestingly, experimental studies have shown that supplementation with betaine can protect against kidney damage, as well as reduce inflammation and decrease the progression of kidney disease^63–65^. One study showed feeding cats with chronic kidney disease food supplemented with betaine and prebiotics increased total body mass and reduced uremic toxins^66^. Therefore, the increased plasma betaine at diagnosis in cats with CE that are dietary responders in the Bristol cohort may offer some protection from kidney disease, which may explain why these cats did not have urinary metabolic changes of the gut kidney-axis similar to the cats in the RVC cohort and why their disease severity scores were lower.

Although glycerol has been shown to ameliorate colitis in animal models^57, 67^, plasma glycerol is increased in Crohn’s disease patients and correlates with the inflammatory biomarker, C-reactive protein^68^. Therefore, for the cats in our study, glycerol could be the cause or contributor to CE, rather than representing a compensatory response to inflammation. Whether exogenous glycerol from the diet fed at diagnosis in these cats could be a cause for the increased plasma levels of glycerol and subsequent intestinal inflammation and therefore, by discontinuing the diet remission can be achieved requires further investigation.

Our study had the following limitations: although cats in the control group did not have any reported GI signs, full physical examination and laboratory blood work was not performed to ensure subclinical GI disease was not present nor was a full history collected to rule out other diseases or current medications. Additional environmental factors such as diet and housing, which are known to affect the biochemical profile^69, 70^ were not controlled for, as this study intended to identify clinically relevant changes in the plasma, urinary and fecal metabolomes of cats with CE compared to cats with non-GI signs and attempting to control for these additional factors would have likely excluded the majority of cats. As such, it may not have been applicable to a clinical setting where cats exposed to a range of environmental factors are seen. In our study, not all cats had GI biopsies performed to confirm an underlying diagnosis of chronic inflammatory enteropathy. Although one cat was excluded due to alimentary small cell lymphoma, additional cats may have been excluded if they all had this procedure performed. However, the main aim of our study was to characterize the metabolomic profile of cats with FRE, and therefore it was considered unlikely that undiagnosed small cell lymphoma was common in this group. However, our broad inclusion criteria would have resulted in our non-FRE group being very heterogenous. Unfortunately, longer term follow-up was unavailable for cats with CE to confirm sustained remission with dietary therapy and to rule out spontaneous recovery over the 6-week period independent of the diet. A final and important limitation of our study was the small number of cats that were included in the CE and control groups. However, untargeted analysis, which was used in our study generally uses smaller sample sizes with larger sample sizes then being employed for targeted analysis.

## Conclusion

This is the first study describing the broad metabolic derangements induced by CE in cats. CE was found to disrupt energy and amino acid metabolism, and such changes were similar to those seen in humans and animal models of IBD. Further work is needed to determine the underlying mechanism of these changes, their functional significance, and to assess whether these signals could act as biomarkers of disease and clinical severity. Extending our understanding of this disorder will assist in the development of therapeutic targets for cats with CE and may also increase our ability to treat humans affected by similar conditions. Finally, our results suggest that urinary or plasma biomarkers at diagnosis can predict whether cats with CE will respond to intervention with a hydrolyzed protein diet. However, due to the low number of cats in each of the responder and non-responder groups, these findings are preliminary and require further studies utilizing larger number of cats for corroboration.

### Endnotes

*Royal Canin Veterinary Diet Feline Hypoallergenic dry food (composition: rice, hydrolysed soya protein isolate, animal fats, vegetable fibers, minerals, hydrolysed poultry liver, soya oil, beet pulp, fish oil, fructo-oligosaccharides, borage oil, marigold extract (source of lutein). Key values per 100 g as fed: protein 25.5 g, fat 20 g, carbohydrate 34.5 g, dietary fiber 8.2 g, metabolizable energy 410 kcal).

### Supplementary Information

Supplementary Information.

## Data Availability

All data generated or analyzed during this study are included in this published article and its supplementary information files.

## Abbreviations

ALP: Alkaline phosphatase
ALT: Alanine aminotransferase
CE: Chronic enteropathy
FCEAI: Feline chronic enteropathy activity index
FRE: Food-responsive enteropathy
GI: Gastrointestinal
IBD: Inflammatory bowel disease
PLI: Pancreatic lipase immunoreactivity
RVC: Royal Veterinary College
T4: Thyroxine
TLI: Trypsin-like immunoreactivity
NMR: Nuclear magnetic resonance
TSP: 3-Trimethylsilyl-1-[2,2,3,3-2H4] propionate
CPMG: Carr-Purcell-Meiboom-Gill
OPLS-DA: Orthogonal projection to latent structures-discriminant analysis
PCA: Principal components analysis
NMND: *N*-Methylnicotinamide
PAG: Phenylacetylglutamine
DMG: Dimethylglycine
pCS: *p*-Cresyl-sulfate
