# Supplementary material for: The effects of a hydrolyzed protein diet on the plasma, fecal and urine metabolome in cats with chronic enteropathy
Source: Sci Rep. 2023 Nov 15;13:19979. doi: 10.1038/s41598-023-47334-y (PMC10652014; doi:10.1038/s41598-023-47334-y)

**Supplementary Figure 1:** Principal components analysis (PCA) model comparing the urinary metabolic profiles of control cats and those with chronic enteropathy (CE) recruited from the Royal Veterinary College (RVC). Scores plot from the model showing principal components (PC) 1 and 2, accounting for 31.8% of the variation in the urinary data. The control cats are represented as black dots and the CE cats are shown as red dots. No separation between the groups can be observed.


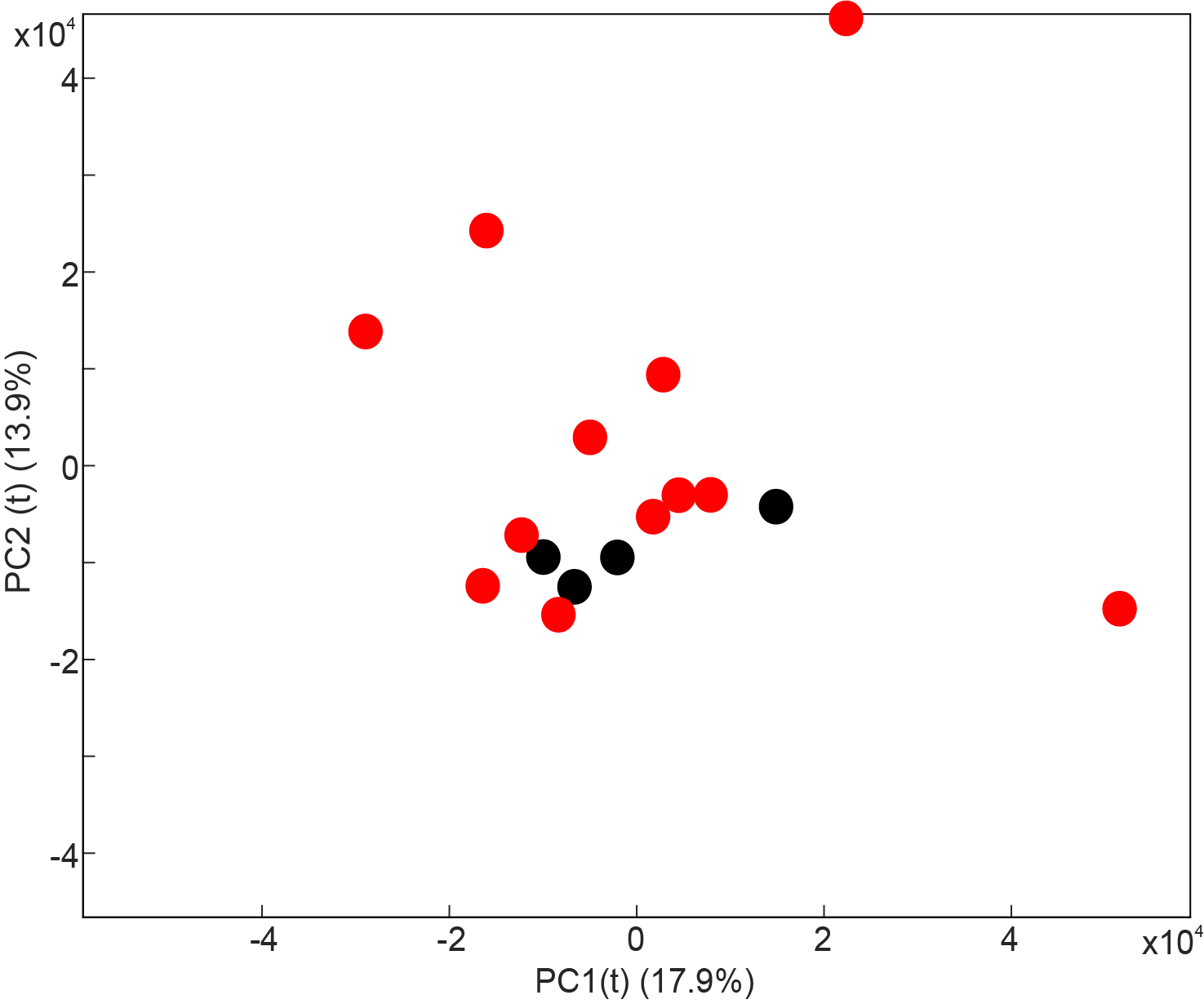


**Supplementary Figure 2**: A) OPLS-DA model comparing the urinary metabolic profiles pre- versus post-diet from the University of Bristol cohort (CE cats pre-diet (*n* = 20), CE cats post-diet (*n* = 12)). Model diagnostics, Q^2^Y = 0.337; *p* = 0.002. Red peaks indicate those with a significant correlation (*p* < 0.05) with sampling point. B) OPLS-DA model comparing the pre- versus post-diet fecal samples from the University of Bristol cohort (CE cats pre-diet (*n* = 22), CE cats post-diet (*n* = 12)). Model diagnostics, Q^2^Y = 0.206; *p* = 0.001. Red peaks indicate those with a significant correlation (*p* < 0.05) with sampling point.


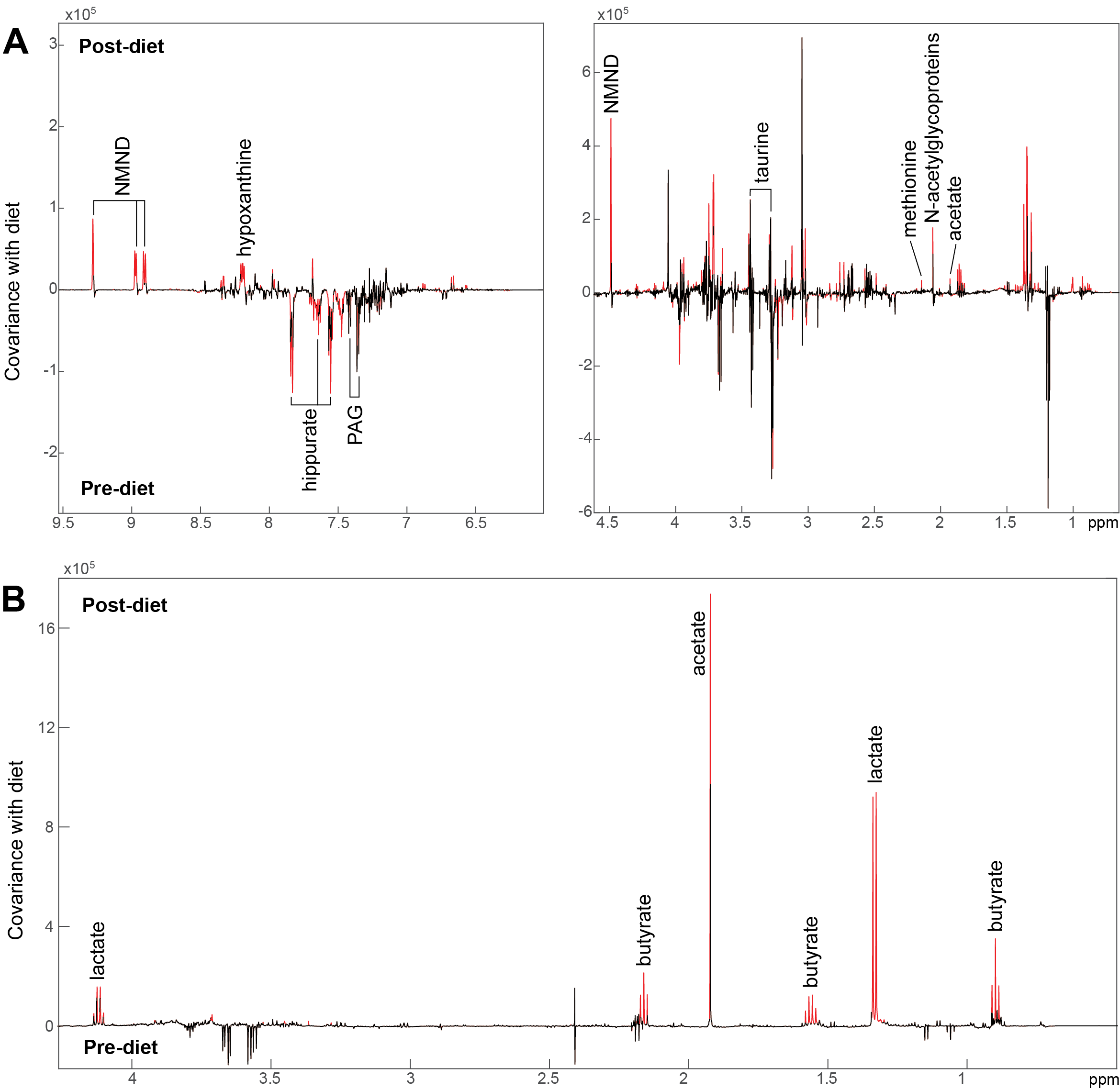

Supplement: Supplementary file 1 — Supplementary Information. [file 41598_2023_47334_MOESM1_ESM.docx]
